# The Contributions of Image Content and Behavioral Relevancy to Overt Attention

**DOI:** 10.1371/journal.pone.0093254

**Published:** 2014-04-15

**Authors:** Selim Onat, Alper Açık, Frank Schumann, Peter König

**Affiliations:** Institute of Cognitive Science, University of Osnabrück, Osnabrück, Germany; University Medical Center Goettingen, Germany

## Abstract

During free-viewing of natural scenes, eye movements are guided by bottom-up factors inherent to the stimulus, as well as top-down factors inherent to the observer. The question of how these two different sources of information interact and contribute to fixation behavior has recently received a lot of attention. Here, a battery of 15 visual stimulus features was used to quantify the contribution of stimulus properties during free-viewing of 4 different categories of images (Natural, Urban, Fractal and Pink Noise). Behaviorally relevant information was estimated in the form of topographical interestingness maps by asking an independent set of subjects to click at image regions that they subjectively found most interesting. Using a Bayesian scheme, we computed saliency functions that described the probability of a given feature to be fixated. In the case of stimulus features, the precise shape of the saliency functions was strongly dependent upon image category and overall the saliency associated with these features was generally weak. When testing multiple features jointly, a linear additive integration model of individual saliencies performed satisfactorily. We found that the saliency associated with interesting locations was much higher than any low-level image feature and any pair-wise combination thereof. Furthermore, the low-level image features were found to be maximally salient at those locations that had already high interestingness ratings. Temporal analysis showed that regions with high interestingness ratings were fixated as early as the third fixation following stimulus onset. Paralleling these findings, fixation durations were found to be dependent mainly on interestingness ratings and to a lesser extent on the low-level image features. Our results suggest that both low- and high-level sources of information play a significant role during exploration of complex scenes with behaviorally relevant information being more effective compared to stimulus features.

## Introduction

The allocation of attention under natural viewing conditions is a complex phenomenon requiring the concerted activity of multiple neuronal levels, mobilizing a huge number of sensory and motor areas as well as subcortical structures. The most straightforward behavioral measure of attentional allocation under natural conditions is given by the subject’s eye movements. Indeed we move our eyes nearly effortlessly and mostly unconsciously while exploring the world. Not surprisingly, eye movements have been in the focus of scientific investigation for decades [1]–[3]. Up until today many different theories have been put forward, which approach the question from different directions [4].

Several independent factors operating in parallel interact and add considerable complexity to the study and generation of eye movements under natural conditions. These include stimulus properties, the relevance of the information for the human observer and geometrical aspects [5]. The first two sources of information are roughly referred to as bottom-up and top-down allocation in the literature. The first conceptualization, namely bottom-up or stimulus-dependent vision, exclusively considers the information content embedded in the stimulus itself. This typically spans a large spectrum, covering local features from a simple (such as orientation, luminance contrast, disparity) to complex level (faces, cars, objects, body parts), but also more distributed features such as symmetry and arrangement of objects. This wide spectrum can be roughly divided into low-, mid- and high-level information, reflecting roughly the cortical hierarchical organization from primary visual cortex to higher visual areas.

The influence of stimulus features is often captured by the concept of saliency maps. Indeed, many years after their introduction saliency models of overt attention have moved back into the center of interest [6], [7]. Based upon the psychophysical results obtained in the field of visual search [8], the authors introduced the concept of the saliency map to embody a generative model of eye movements for the exploration of more complex, photographic scenes. It attributes a direct role to local image characteristics in the process of oculomotor response generation [6], [7], [9], [10]. This view is supported by the fact that stimulus properties at fixated locations differ significantly from non-fixated locations [11]–[15]. Furthermore, these computations are thought to be mainly externally driven and not task dependent. Hence, the signals of a hypothetical saliency map are relayed in a feed-forward (bottom-up) fashion to motor centers. In sum, the use of saliency is proposed to provide the brain with an efficient and fast method to extract locations of general relevance in an image [16], [17].

In parallel to the bottom-up mechanisms of attentional allocation, the actual intentions of the viewer contribute also to eye movements. In this view, the viewer selects those locations that are behaviorally relevant, either for the task at hand, or more generally, to understand the semantic content of the image. A large body of evidence supports the view that the human visual system has a considerable knowledge about the semantic aspects of the visual scene very early following its onset. For instance, the human visual system is able to recognize the identity of objects shown in a rapid serial fashion [18] even at the peripheral part of the visual field [19]. Even in the near absence of attention, categorization of natural images is fast at high performance levels [20]. Furthermore, second-order statistics of natural images, referring to the relationship between a pair of values, may provide the visual system with necessary contextual information that can be exploited for the guidance of overt attention [21]. It has been shown that these cues are rich enough to potentially direct attentional resources to appropriate locations starting very early with the presentation of stimuli [22]–[24]. For example in an image with a clear horizon line, prior knowledge about where the interesting objects are, could be obtained by these contextual cues without the need of scanning potentially behaviorally irrelevant locations. These findings suggest that the visual system is able to very efficiently extract the behaviorally relevant information from complex natural scenes and use this information to direct attentional resources in a top-down manner.

In this scenario, locations of an image are mostly fixated in order to gather highest-quality visual information on behaviorally relevant parts, using the high spatial resolution of the central visual field. The driving force underlying fixation point selection may be the existence of an explicit task [25] or simply the presence of behaviorally relevant objects in the scene. These include the spatial location [26] and semantic congruency of objects [27]–[29], the informativeness of image locations [5], [30]–[32], multimodal interactions [33], colocalization of auditory and visual stimuli [34], the potential or past reward [35], as well as the associated interestingness of the scene content [36].

However, we have to differentiate between stimulus-dependent high-level features and allocation of attention guided by behavioral relevance. On one hand it is possible to voluntarily direct attention to simple low-level features. Hence, top-down guided attention is not identical to attention of visually complex high-level features. On the other hand high-level features like objecthood can influence the allocation of attention even outside of the context of an explicit task [26], [37]. Thus, it is important not to exclusively equate bottom-up guided attention to simple low-level visual features. For example, a recent report demonstrates a high probability for fixations on objects in a patient suffering from a deficit in object recognition [38]. This suggests that complex stimulus features such as objects can also have a direct bottom-up influence on eye movements despite the patient’s inability to recognize objects. Hence, bottom-up guided attention is not identical to attention of simple low-level visual features. These results highlight the complementarity of bottom-up and top-down attentional systems and their complex, still ill understood interactions.

A limitation of many models of overt visual attention is that they focus primarily on the prediction of fixated positions and neglect an important parameter of eye movements: the duration of fixations [4]. The simple fact that fixation durations can vary significantly suggests that fixations can and should not be treated equally. Instead, they point to the fact that there is an underlying on-going perceptual and/or cognitive process during the analysis of the image. It is not clear to what extent stimulus-dependent aspects and behavioral relevancy modulates fixation durations. Previous reports showed that semantic congruency [4], [28] and informativeness of image ratings are important parameters that modulate fixation durations. Within this scheme, the duration of fixation points could reflect the time that is required to integrate the information present in an image region.

Our working hypothesis is that overt attention is a process whereby humans actively collect information from the external world and try to construct a coherent, meaningful representation. The information collected can be tailored according to the requirements of a given situation or task. However, even in the absence of an explicitly defined task, it can be argued that humans nevertheless try to understand what a scene is about. We therefore excluded any task-specific bias, and aimed to characterize to what extent stimulus-dependent as well as behavioral saliency contribute to attentional allocation processes under default, baseline-viewing conditions. We refer to stimulus-dependent information as any scalar value derived directly from pixel intensities. And this is complemented with behaviorally salient information content, as estimated by the interestingness ratings given by human subjects (similar to [36]). In the present study, we aimed to quantify to what extent stimulus-dependent and behaviorally relevant information account for the observed eye movements and how these different sources of information are integrated for overt attention.

## Materials and Methods

### Experimental Paradigm and Stimulus

We recorded eye movements of 48 subjects (25 males, mean age 23.14, range 19–28), who were naive to the purpose of the experiment. They were either accredited for 1 hour of research participation or paid 5 Euros. The participants were instructed to study the images carefully. All participants gave informed written consent at the start of the experiment. All experimental procedures were in compliance with guidelines described in Declaration of Helsinki and approved by the ethics committee of the University of Osnabrück.

Four categories of colored images belonging to the categories of natural, urban, fractal and pink noise were presented. Images of the natural category (selected from McGill Calibrated Color Image Database) excluded any kind of human artifacts. Photographs of bushes, trees, forests and flowers were typical of this category. Photographs making up the urban category were taken with a high-resolution camera (Nikon D2X, Tokyo, Japan) at public places in and around Zürich, Switzerland. These were characterized by cityscapes where man-made artifacts, vehicles, buildings, humans, scripts and streets were common. Fractal images were obtained from three different web databases (Elena Fractal Gallery, http://web.archive.org/web/20071224105354/http://www.elena-fractals.it/; Maria’s Fractal Explorer Gallery, http://www.mariagrist.net/fegal; Chaotic N-Space Network, http://www.cnspace.net/html/fractals.html). Uncompressed images were requested from the authors of the corresponding websites. We took care that the axis of symmetry of fractals were not always overlapping with the middle line of the monitor. To generate pink noise images, we computed the amplitude spectra of each single stimulus keeping separate the RGB color channel. This was done by transforming the original pixel space into frequency domain using discrete Fourier transformation. To obtain category specific average amplitude spectra, these were averaged across all images being member of a given category. To generate single pink noise images, these category specific amplitude spectra were then combined with a random phase spectra and transformed back to pixel space for each color channel separately with discrete inverse Fourier Transform. The random phase spectra were obtained using Fourier transformation of a random white noise image and discarding the amplitude information. This effectively took care of the symmetrical organization of the phase spectra. All categories contained 64 images except the pink noise categories that contained 63 images (21 for each category) making 255 images in total.

The images were presented on a calibrated 21-inch Samsung SyncMaster 1100 DF 2004 CRT Monitor (Samsung Electronics Co, Ltd., Korea). The display resolution was set to 1280×960 (4∶3) with a refresh rate of 85 Hz. Subjects sat on a stable chair, in 80 cm distance to the screen. The center of the screen was approximately at the eye level for all subjects.

Eye movements were recorded using the video-based EyeLink II system (SR Research Ltd., Mississauga, Ontario, Canada). Before the experiment, a 3×3 calibration grid and validation procedure was applied until desired calibration quality was obtained. This procedure lasted for several minutes, thus allowing subjects to adapt to the conditions within the experimental room. The image presentation and eye tracking started only after the absolute mean calibration error was below 0.3° for at least one eye.

Fixations were defined using the default settings of the eye-link tracker. We defined fixation points and intervening saccades using a set of heuristics. A saccade was characterized by an acceleration exceeding 8000°/s^2^, a velocity above 30°/s, a motion threshold of 0.1°, and a duration of more than 4 ms. Intervening episodes were defined as fixation events. The result of applying these parameters was plotted and was visually assessed to check that they produce reasonable results.

Each image was shown for 6 seconds and the order of presentation was randomized for each subject. Each subject performed only one session that lasted less than an hour. A break was introduced after the first half of the stimulus set had been displayed. The eye-tracker was removed from the participants’ head if they wished to. In those rare instances the calibration procedure was carried out anew as described above. During the experiment, drift errors were corrected via a fixation point that appeared in the center of the screen before each stimulus presentation. If drift errors were high, the eye-tracker was recalibrated.

### Computation of Feature Maps

For analysis of low-level feature values at the fixated locations stimuli were converted into the DKL color space [39]. This color representation is based on the fact that there are three different cone types in the retina, namely short (S), medium (M), and large (L), which have different wavelength absorbance spectra. In the DKL space, two color channels are constructed with L and M channels (Red-Green channel) and S and (L+M) channels (Blue-Yellow channel). The third channel constructed with (L+M+S) represents the brightness of the stimuli. All the feature maps were computed based on these 3 channels.

**Figure 1:**
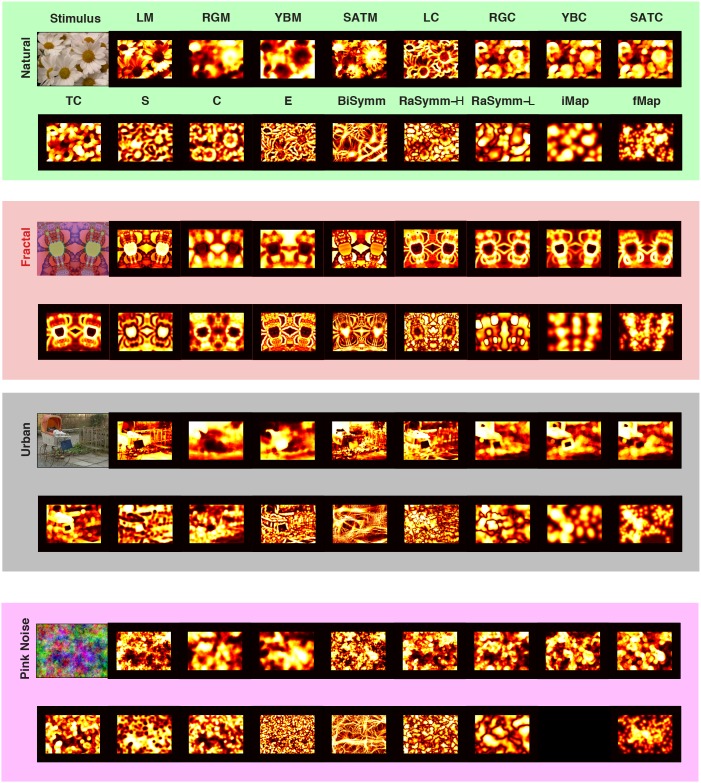
Decomposition of stimuli into a set of low- and high-level features. For each category (*first* row, *green* background, **Natural**; *second* row, *red* background, **Fractal**; *third* row, *black* background, **Urban**; *last* row, *magenta* background; **Pink Noise**) a representative stimulus (left upper corner) and its associated low-level feature maps are shown. **LM**: Mean Luminance; **RGM**: Mean Red-Green Intensity; **YBM**: Mean Yellow-Blue Intensity; **SATM**: Mean Saturation; **LC**: Luminance Contrast; **RGB**: Red-Green Contrast; **YBC**: Yellow-Blue Contrast; **SATC**: Saturation Contrast; **TC**: Texture Contrast; **S**: Surfaceness; **C**: Cornerness; **E**: Edgeness; **BiSymm**: Bilateral Symmetry; **Ra-Symm L/H**: Radial symmetry with high or low spatial frequency selectivity. In addition to these low-level features, interestingness ratings were collected with the help of a pointer device (see Material and Methods), the topographic distribution of this high-level feature is shown as interestingness maps (**iMap**: Interestingness Map). Please note that this data is not collected for the case of Pink Noise category (lowest row). In addition to click data, recorded eye-movements for these four images are also presented in the same topographic form (**fMap**: Fixation Map; *second* row in each panel, *last* entry). All these maps were are shown following the histogram equalization step therefore all values occur equally likely.

To investigate the relationship between low-level image features and fixation locations, we computed an extensive set of feature maps (Fig. 1). In doing so, we used 3 different mutually exclusive classes of low-level features: Features that selectively sensitive to first- and second order characteristics of feature values as well as features that were selective to the configurational aspects of the local image content. First-order features capture the local average intensity of channel values and these included Mean Luminance intensity (**ML**), Color Intensity for Red-Green and Yellow-Blue channels (**RGM** and **YBM**) and Saturation (**Sat**). Second-order features were used to measure the spread of the channel values distributions and hence these quantify the local contrast. These were Luminance Contrast (**LC**), Red-Green Contrast (**RGC**), Yellow-Blue Contrast (**YBC**), Saturation Contrast (**SatC**) and Texture Contrast (**TC**). We computed configuration specific features to characterize the content of local patches of images. Importantly these features are independent of the above-cited features. We computed cornerness (**C**), edgeness (**E**), surfaceness (**S**), bilateral and radial symmetry (**SymB, SymR**) features. These maps were created for each single stimulus based on the information present at different DKL channels by computing the value of a given visual feature within a small circular aperture for each possible location on the image. Window sizes were specific for each feature and we chose the one that gave the best results in terms of the strength of the correlation between fixation positions and features values. The size of windows was kept constant for all categories of images.

Mean Luminance feature was computed using pixel values in the brightness channel by averaging all the pixels within a circular patch of 0.5° diameter. Red-Green and Yellow-Blue features were computed similarly using the RG and YB channels (diameter = 2°), respectively. Within these two feature maps, high intensity represents Red/Yellow hue and low values the Green/Blue hues. Saturation feature was created by taking the square root of the squared sums of each individual color channel as follows: 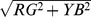. The mean saturation feature was created by computing the average saturation in a local patch of 1° diameter.

All second-order features, Luminance Contrast, Saturation Contrast (diameter = 1°), Red-Green Contrast and Yellow-Blue Contrast (diameter = 2°) were computed by taking the standard deviation of pixel values within corresponding channels. The texture contrast feature is defined as the contrast of contrast values. We therefore computed Texture Contrast maps by computing the standard deviation of contrast values in a patch of diameter 3.7° in a previously computed contrast map.

Configuration selective features of Cornerness, Edgeness and Surfaceness were derived by computing the Intrinsic Dimensionality of local image patches ([40], see definition in [41]). Intrinsic dimensionality characterizes a given image patch according to the number of dominant orientations present. For example, patches with high intrinsic dimensionality of level one are defined by a single dominant orientation. Similarly intrinsic dimensionality of degree two scores high when the patch contains two dominant orientations characteristic of corners, junctions and crosses. We computed the intrinsic dimensionality of degree zero (Surfaceness), one (Edgeness) and two (Cornerness) within Gaussian patches of 6° with a standard deviation of 1°. The images were smoothened slightly with a Gaussian Kernel of a standard deviation of 0.13°. Importantly intrinsic dimensionality operates independent of the above features; therefore the detection performance is not influenced by the luminance contrast or average intensity.

The contribution of symmetrical configurations was characterized using Phase Symmetry feature developed by Peter Kovesi [42], [43]. These features quantify each image location according to phase relationship of different components in the Fourier space and measure the strength of radial and bilateral symmetrical configurations in an image locally.

As a next step we gauged the performance of the above-cited feature maps in predicting the fixation behavior. If a given feature map predicts perfectly the fixation behavior it should be similar to the fixation behavior of the subjects and thus it should reflect for each location in an image the probability of being fixated. Hence, we made use of the empirical fixation maps and pooled all the fixation points done on a given image by all subjects and computed the fixation maps (see next section for details).

It is important to note that the distribution of feature values in different features maps may well have different global properties. For example, the distribution of luminance contrast values can have different second- or higher-order characteristics than the distribution of another feature channels. As we wanted to test here the saliency of many different features it is important that the lower and higher moments of feature distributions are controlled. Furthermore, distributions of feature values are typically heavy tailed, making the estimation of saliency at these points difficult. We thus used a histogram equalization scheme in order to transform absolute feature values into percentiles. This effectively removed all differences between distributions of different features.

### Image and Category Specific Fixation Maps

The term *image specific fixation map* (or actual fixation map), FM*_i_*, refers to the spatial distribution of fixations points made by all subjects on image, *i*. These maps reflect the probability of each location in a given specific image to be fixated and therefore they represent the actual behavior of subjects when viewing an image. *Category specific fixations maps* (or control fixation map) are computed by pooling all fixations done on all images belonging to a given category, <FM>*_i_*, where <>*_i_* represents the average across individual images belonging to a given category of images. The distribution of fixation points on these maps reflects image-unspecific behavior of subjects resulting from different biases on the fixation behavior such as for example central bias [44], [45]. These maps represent the global behavior of subjects when different categories of images are viewed. These two types of fixation maps were created by collecting spatially all the fixation events and smoothening these with a Gaussian kernel with 1° of full-width at half maximum.

We treated these maps as probability distributions and computed their entropy using the following formula, where H(FM*_i_*) is the entropy associated to the i^th^ fixation map, P_i_(x) is the fixation map with unit integral:
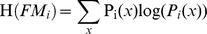



In the case of actual maps, the entropy values were averaged across different images of the same category, after having computed the entropy of the individual fixation maps, <H(FM*_i_*)>*_i_*, where H(FM*_i_*) represents the entropy of the actual fixation map of image *i.* We also computed the entropy of the control distribution for each category, H(<FM>*_i_*). As the absolute values of the entropy depend on the precise binning of these maps, they are irrelevant for comparison; we thus normalized these with the maximum theoretical entropy value (uniform distribution) obtained under same binning conditions. As the number of fixations that contributed to this analysis was high enough we didn’t need to control for the intrinsic bias that occurs with small number of fixations [45].

### Bayesian Framework for Deriving Saliency Functions

In this report we concentrate on free viewing conditions. This implies that fixations are not driven by a specific task, but by stimulus-dependent effects. We do not differentiate probability vs. saliency and use the terms “fixation probability” and “saliency” equivalently. Hence a location in an image that has a high probability of fixation is considered to be a salient location. For each single image, *i*, and single subject, *s*, we quantified the saliency function p(fixation|feature = X) associated to different features using the following Bayesian equality:
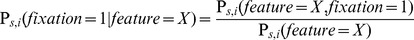
where *X* denotes the feature percentiles and it ranges between 0 and 100, *s* and *i*, represents individual subjects and images. p*_s,i_*(feature = *X*) represents the distribution of features in a given image and is a constant function of X due to the histogram equalization process. Fixation = 1 indicates the occurrence of a fixation at that location. Fixation = 0 indicates the absence thereof.

During the histogram equalization process, we took into account the central bias of fixation points [12], [44] and spatially weighted feature values with each subjects’ category specific fixation distributions (control distributions). This is necessary in order to take into account the strong central bias in the scanning behavior of subjects. In case a photographer or experimenter bias leads to an inhomegeneous distribution of image features across space, the central bias in the viewing behavior may result in a bias of the feature values at fixation locations. We therefore weighted feature values depending on their positions in the image using the control fixation maps. Therefore, following the histogram equalization and weighting process, the distribution of feature values at all fixated locations, p*_s,i_*(feature = X), became a uniform function of feature percentile. This distribution represents the values of features at all fixated locations including actual and control fixations. Importantly, this histogram equalization procedure was carried out separately for each single image and subject.

The joint distribution p*_s,i_*(feature = X, fixation = 1) represents the probability of features at fixated regions. It was computed by evaluating the probability of feature percentiles at actually fixated locations. The ratio of these two terms is equal to the saliency function p*_s,i_*(fixation = 1|feature = X), that is the probability of a given feature value to be fixated. The constant, p(fixation) is the probability of a fixation point to be the actual fixation. As it consists of a constant we can ignore this term. The distributions were subsequently averaged across all subjects and images that belonged to a given category. The Matlab toolbox that was used to carry out this analysis is available at https://github.com/selimonat/published_code/tree/master/condprob.

In order to quantify the contribution of different features to fixation behavior we quantified the strength of the correlation between fixation points and feature values. If fixation behavior during viewing of an image is not guided by the low-level features content, one would expect that the distribution of feature values at fixated locations is a random sample of the distribution of feature values overall. Therefore, any correlation that exists between feature values and fixation behavior would result in deviations between the saliency function, p(fixation = 1|feature), and the image statistics, p(feature), distributions. Consequently, the strength of the correlation between overt behavior and low-level feature values can be characterized by measuring these deviations. Kullback-Leibler divergence, D_KL_, captures any deviations present between two distributions thus can be used to quantify the strength of the correlation.
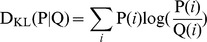
where P and Q represent feature distributions at control and actual locations. However, D_KL_ measure is not a symmetric metric to measure similarities so that D_KL_(P|Q) is not equal to D_KL_(Q|P). We therefore used a symmetric version of D_KL_ using the following formula,



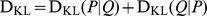



One of the benefits in using information theory derived measures, such as D_KL_, over more traditional signal detection measurement tools, is that within the Bayesian framework we are using, the generalization of D_KL_ values to more than one feature dimensions is straightforward. Furthermore, D_KL_ measure is sensitive not only to linear correlations but to any kind of correlations and deviations from independence of probability distributions.

We quantified for each subject the D_KL_ values associated with different features and categories. Differences in the average D_KL_ values were evaluated using ANOVA test with repeated measures using categories, and features as main factors. This was done after log transformation of D_KL_ values, which effectively normalized these distributions. We applied Lillie test to evaluate the normality of log transformed D_KL_ measurements. In all the feature-category pairs, the log transformed D_KL_ values were found to not significantly deviate from a Gaussian distribution (p>0.01). Confidence intervals for D_KL_ values were computed using boot-strap method, where the distribution of average D_KL_ values were obtained selecting 1000 times with replacement from the pool of D_KL_ values where each entry was specific to a given subject.

### Interestingness Maps

In order to gather high-level information associated with different locations in an image, we asked another set of (n = 35) subjects to click with the help of pointer device on locations of an image that they found subjectively interesting. The fact that a different cohort of subjects performed the interestingness evaluation potentially introduces intersubject variations. However, we have no indication of a significant difference between the two groups with respect to parameters age and gender. Furthermore, it is highly preferable that subjects do not view the stimuli more than once, as repeated presentations of the same stimulus material introduces systematic biases [46]. Furthermore, the interindividual effects are typically small and require careful experimental design to be demonstrated [47]. The probability that unrecognized systematic differences between the two large cohorts exist can be considered negligible. Finally, the experimental procedure and design were kept as similar as possible to the eye-tracking experiment. The experiment took place in the same room using the same monitor for image display. They were shown the same images as in the eye-tracking study and required to select 5 points, which they found subjectively most interesting. Subjects were instructed to scan the totality of the image before making any decisions on the interestingness rating. The experiment was self-paced so that there was no time pressure on the subjects and the images stayed on the screen as long as it took for subjects to select 5 interesting points.

Similar to low- and mid- level feature maps, we created interestingness maps by pooling all the clicks on an image done by all the subjects and smoothening these with a Gaussian kernel of 2°.

## Results

### Exploration Strategies

We recorded eye movements of human subjects (n = 48) while they were freely viewing photographs of natural and urban scenes as well as complex artificial patterns. We used 4 different categories (**N**atural, **F**ractal, **U**rban and **P**ink Noise, see Fig. 1) each containing 64 images, except Pink Noise category that contained 63 images. Using a Bayesian framework, we quantified to what extent eye movements made by human subjects correlate with low-level image characteristics that are presumably extracted during sensory processing in the brain. Furthermore, we evaluated how different bottom-up information channels in isolation or in pairs are integrated into behavioral saliency and subsequently compared the saliency of these bottom-up channels to the saliency of high-level characteristics of the images, as provided by the interestingness maps. Furthermore, we evaluated how low-level and high-level information are integrated in the generation of eye-movements.

First, in order to justify the selection of our image categories and understand better how the exploration strategies differed among these categories, we investigated image specific (64 maps for each category) and category specific (one map for each category) fixation maps. We treated these maps as probability distributions and computed their entropy (see Materials and Methods) in order to evaluate the spatial correlation between fixation positions at the *image*- and *category*-level. Whereas high entropy values signal uniformly distributed fixation points across space, low values indicate highly structured maps due to accumulation of fixation points at similar locations. Therefore in the case of *actual* maps (image specific fixation maps), the entropy characterizes the inter-subject similarity, low entropy values notify high inter-subject agreement. In the case of *control* maps (category specific fixation maps) entropy measures the inter-image similarity. Here high entropy result when different images of the same category induce similar fixation patterns.

**Figure 2:**
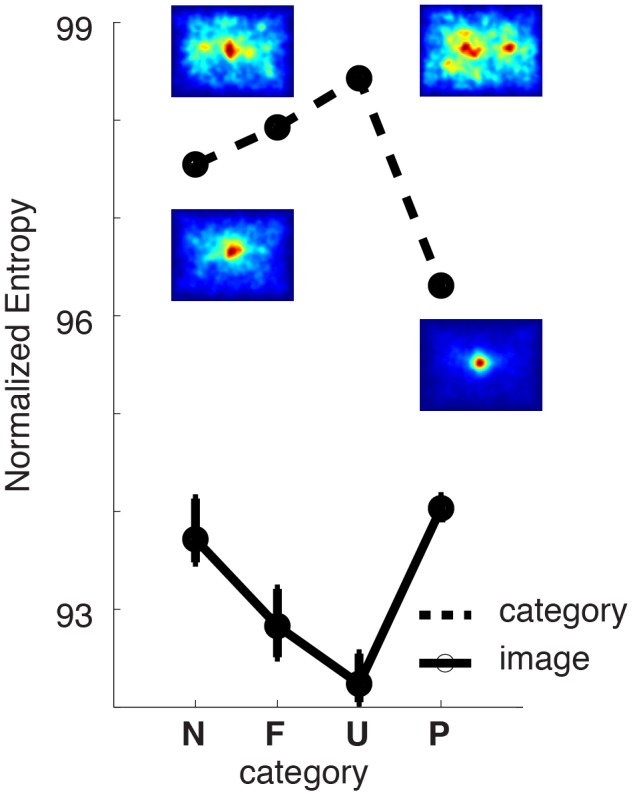
Exploration strategies. Entropy values for category (*dashed* line) and image (*solid* line) specific fixation maps for each stimulus category (here and in the following Figures, **N**: Natural; **F**: Fractal; **U**: Urban; **P**: Pink Noise). In the case of image specific fixation maps, the average entropy across all images belonging to a given category is shown. Error bars represents 99% bootstrap confidence intervals. Inset maps depict category specific fixation maps that represent the distribution of all fixation points across all images and subjects.

Overall the entropy of control maps was about 5% higher than image specific fixation maps. This is expected given that control maps are much more uniformly distributed, as they result from the average behavior where the effect of individual images is washed out. Across categories we found a significant main effect of image category (F(3,251) = 21.91, p = 1.2×10^−12^, ANOVA). The category of Urban images lead to highest category specific entropy (Fig. 2, *dashed* line) and smallest image specific entropy (Fig. 2, *solid* line) values. This shows that the viewing behavior of each single image was characterized by a high inter-subject agreement. At the category level, however, this behavior leads to an effective covering of the whole visual area and thus low inter-image similarity. There was a significant difference between image specific entropy values of Fractal and Urban image categories (two-sample t(126) = 1.98, p = 0.049). Interestingly the image specific fixation maps in the case of Pink noise images had similar entropy value as for natural images (two-sample t(126) = −1.65, p = 0.101). This suggests that the total coverage of the stimulus area on an individual image basis was similar between these categories. However the category specific entropy values in the case of Pink Noise images were much smaller than Naturals. This suggests that while the total coverage on an individual image basis was the same between these two categories, subjects tended to look at similar locations during viewing of different pink noise images leading to high inter-image correlations.

Overall, these results justify our selection of categories in behavioral grounds given that most categories induce different exploratory behaviors. Furthermore, we show that while Urban and Fractal category lead to highest inter-subject agreement, Pink Noise and Natural images lead to highest inter-image agreement. It is also important to note that although Pink Noise images are devoid of any higher order correlations, they were scanned in a similar fashion as images belonging to the Natural category.

### Saliency Functions of Stimulus-dependent Features

As stated earlier, bottom-up models of overt attention attribute an important role to low-level image features in the process of fixation point selection. It is indeed a well-established fact that fixation points are preferentially directed at locations that have different feature statistics than non-fixated locations [11]–[15]. For example, it has been consistently observed that fixated locations are characterized with higher luminance contrast values. This consequently led to the claim that image locations with elevated contrast are salient in the sense that they increase the probability of attracting eye-movements. However the precise dependence of fixation probability on feature levels i.e. feature specific saliency function, is generally not explicitly investigated.

To elucidate how saliency changes with different feature percentiles, we used a Bayesian framework. We derived the saliency function, p(*fixation*|*feature* = X) from the two terms of the Bayesian equality, p(*feature* = X) and p(*feature* = X|*fixation*). P(*feature* = X) is the distribution of feature values and therefore describes the image statistics at all locations in an image including both actual and control fixation locations. For a given specific image, feature values at control fixations were obtained using the fixations on all other images of the same category. The term p(*feature* = X|*fixation*) is the distribution of feature values at fixated locations. The ratio of these two terms is equal to the saliency function (see Materials and Methods). These terms were computed for each individual image and subject separately and averaged afterwards. As the image statistics, p(*feature*), was computed based on the distribution of feature values at all fixation points, rather than at randomly selected locations, this method took automatically into account any spatial biases in the distribution of feature values across the image (for example high contrasted regions to be consistently centrally located in our stimulus database). Furthermore, this effectively removed the effect of category specific spatial biases of fixations distributions on the distribution of feature values (see Fig. 1 insets).

As a next step, we included an extensive set of local, low-level visual features that operated on luminance, red-green and yellow-blue channels (Fig. 1). Within these channels we determined the average intensity (**ML**: Mean luminance, **RGM**: Mean Red-Green, **YBM**: Mean Yellow-Blue and Sat: Mean Saturation), the first order contrast (**LC**: Luminance Contrast, **RGC**: Red-Green Contrast, **YBC**: Yellow-Blue Contrast, **SatC**: Saturation Contrast) within small image patches (see Material and Methods for details). Iterating the same computation on the luminance contrast using a larger window resulted in the Texture Contrast. This allowed the detection of changes between regions of images containing different textures assuming they are defined by changes of local luminance contrast [48]. In order to understand the dominant image configuration present in these local patches we computed the Intrinsic Dimensionality of different orders [40] and obtained 3 different complementary features: Surfaceness (**S**), Edgeness (**E**), and Cornerness (**C**). In order to evaluate the saliency of symmetrical configurations, we used Bilateral (**BiSymm**) and Radial Phase Symmetry (**RaSymm-H** and **RaSymm-L** for low and high spatial frequencies) features [43]. These features are all directly derived from the image data and represent stimulus-dependent saliency. Furthermore, given the limited computational complexity we consider them as low-level and mid-level characteristics of the image.

**Figure 3:**
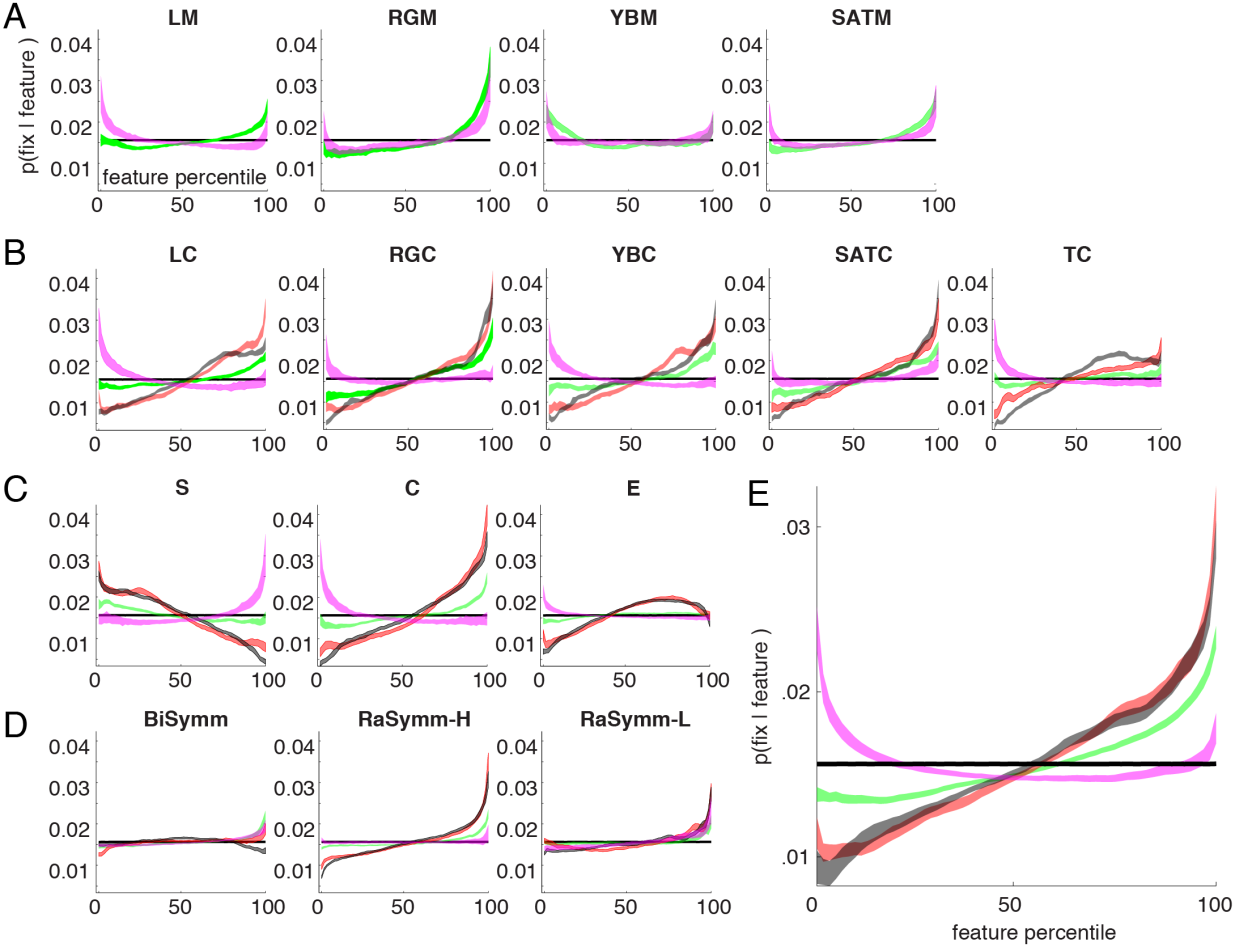
Saliency functions. (**A–D**) Saliency functions, p(fixation|feature), are shown for Natural (*green*), Fractal (*red*), Urban (*black*) and Pink Noise (*magenta*) category of images. Different panels group feature maps according to their selectivity. Features selective for channel intensity (**LM**, **RGM**, **YBM**, **SATM**) and for contrast (**LC**, **RGC**, **YBC**, **SATC**, **TC**) are shown in **A** and **B**, respectively. Only those saliency functions that deviated significantly from the control distribution are shown, notice that saliency functions corresponding to Urban and Fractal categories are omitted in panel A. Last two rows (**C–D**) depict the saliency functions for features of intrinsic dimensionality (**S, C and E**) and for symmetry related features (**BiSymm, RaSymm-H/L**). Shaded areas represent 99.99% bootstrap confidence intervals. Same abbreviations as in Fig. 1. Saliency functions averaged across features (see text for details) are shown in (**E**) for each category of stimuli. In all panels, the horizontal line represents the histogram equalized distribution of feature values, p(feature) after correcting for the central bias of category specific fixations maps.

The saliency functions, p(*fixation*|*feature* = X), associated with these 15 visual features and 4 image categories (*green* for Natural, *black* for Urban, *red* for Fractal and *magenta* for Pink Noise) are shown in Fig. 3A–D. Panels pertain to different low-level features and are further grouped according to their sensitivity with respect to image structure. The first row (Fig. 3A) depicts the saliency functions for first-order low-level features, which are mainly sensitive to the local intensity within different channels. Next 3 rows (Fig. 3B–D) regroup features selective for second-order statistics, intrinsic dimensionality and symmetrical configurations respectively. In each panel the constant horizontal line represents the distribution of low-level features at fixated and non-fixated locations, that is p(feature) (*thin black* line in each plot). If the eye movements on an image were not guided by the low-level image content, the distribution, p(fixation|feature) would not systematically deviate from the distribution p(feature). Consequently the saliency function, p(fixation|feature = X), would also be a constant function of different feature values. In the other case, if certain features consistently correlated with fixation positions, this would lead to notable differences between the saliency function and feature distribution.

Only during viewing of Natural and Pink Noise category of images, the statistical distribution of the first-order features (Mean luminance, Mean Red-Green, Mean Yellow-Blue and Mean Saturation) at fixated locations differed from those of non-fixated locations (Fig. 3A *green* and *magenta* lines, the width of the lines represents bootstrap derived 95% confidence intervals). Analysis of the saliency function of Mean luminance feature in the natural conditions (Fig. 3A, green line, leftmost panel) shows that the probability of fixation points to be directed on brighter image locations was higher than darker locations and the saliency function nearly always monotonically increased with feature intensity. Interestingly we observed the opposite trend in the case of Pink Noise category (*magenta* line) where fixations targeted preferentially darker spots with a small bias on the very brightest locations. Saliency curves for mean saturation (Fig. 3A, rightmost panel) show that in the Natural category, fixation positions were preferentially made on saturated locations, whereas in the pink noise condition both saturated and unsaturated locations were associated to the attentional allocation. The saliency of color channel intensity (Mean Red-Green and Mean Yellow-Blue) shows that red and blue hues were more likely to be fixated in comparison to green and yellow hues and this pattern was similar between these two categories of images.

The saliency of second-order features (Luminance Contrast, Red-Green Contrast, Yellow-Blue Contrast, Saturation Contrast and Texture Contrast) revealed that in all categories the statistical distribution of feature values differed at fixated locations (Fig. 3B, second row). The saliency functions were generally steeper compared to the average intensity features. And in all categories but in the case of Pink Noise the saliency monotonically increased with feature level suggesting that different categories may have different saliency functions.

Concerning intrinsic dimensionality features, only in the case of Cornerness feature a relationship between feature level and fixation probability increased monotonically (Fig. 3C). Here, the slope was biggest in the case of Fractal and Urban category. This shows that the fixation points targeted those regions that are best described by the presence of more than one dominant orientation such as corners, crosses etc. Furthermore, the monotonously declining saliency functions in the case of Surfaceness feature indicates that the fixated locations are rarely characterized by flat surfaces. These two observations explain why the image locations defined purely by a single orientation do not have strong saliencies because of the interdependency between different intrinsic dimensionality features. Importantly, within these intrinsic dimensionality features, the category of Pink Noise differed again from the other three categories with respect to the shape of the slope of saliency curve. For example, whereas during Natural, Urban and Fractal categories, fixations were repulsed from the homogenous locations; those locations were more likely to be fixated during pink noise conditions.

Among the three features that are sensitive to different kinds of symmetrical configurations (Fig. 3D), we found that Radial Symmetry features were more salient than Bilateral Symmetry features and fixation points were preferentially located at image locations that had a high radial symmetrical configuration. This effect was especially strongest for Radial Symmetry tuned to higher spatial frequencies, where strongest slopes were observed for Urban and Fractal categories of images.

### Quantification of Saliency Functions

The saliency functions for each feature and category were evaluated by the strength of the correlation between features and the location of fixation points, quantified by a symmetric version of the information theory based Kullback-Leibler divergence (D_KL_) metric (see Materials and Methods). A larger D_KL_ value indicates a bigger deviation between feature statistics at fixated and non-fixated locations. We computed 60 D_KL_ values corresponding to 4 categories and 15 visual features for each individual subject.

**Figure 4:**
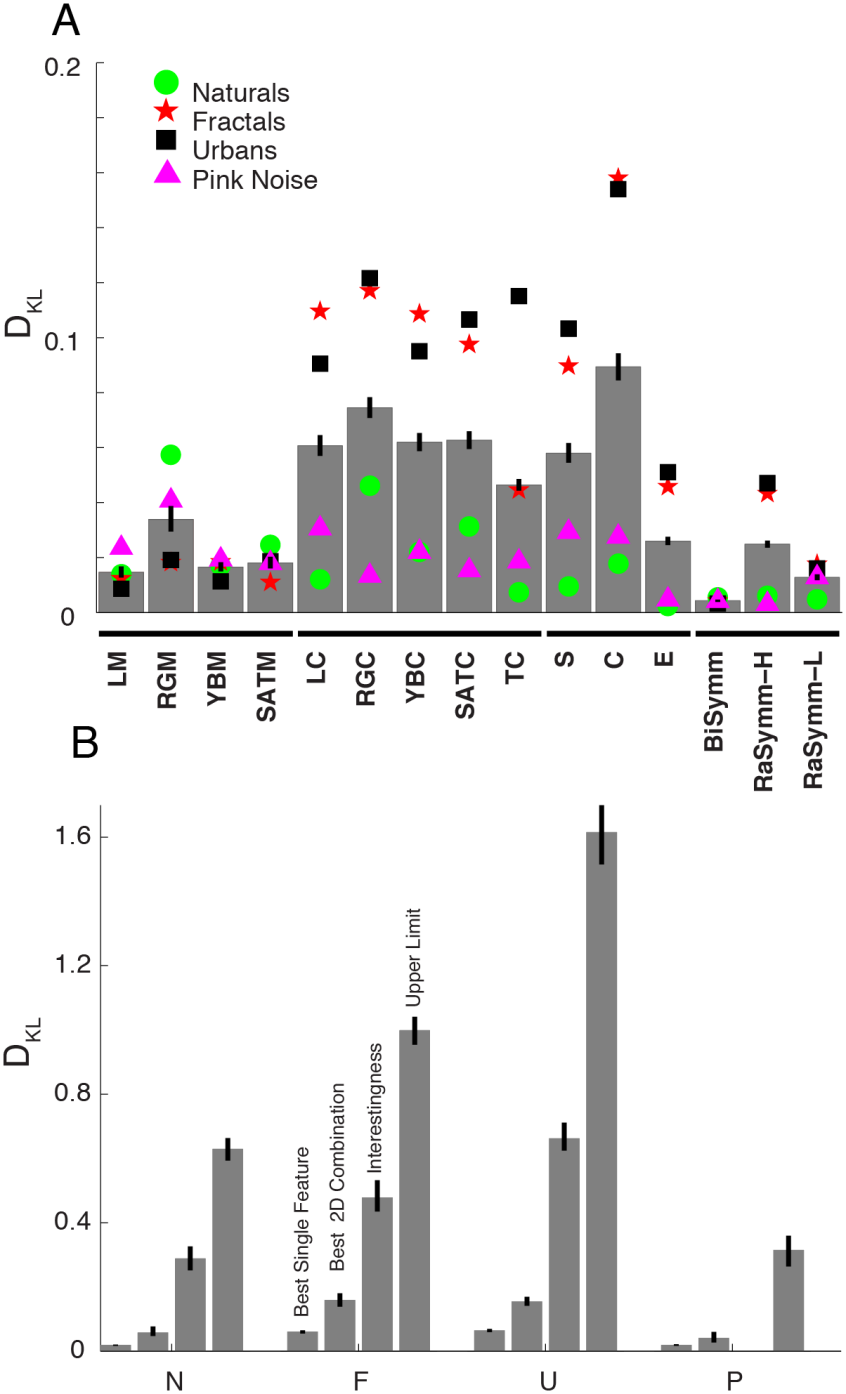
The strength of correlation between low-level features and fixation points. (**A**) Each bar represents the strength of correlation between fixation and low-level feature values quantified with D_KL_ metric and averaged across all categories. For each feature channel, four symbols show additionally the stimulus-category specific D_KL_ values (*green circle*: Naturals; *red star*: Fractal; *black square*: Urban; *pink triangle*: Pink Noise). Horizontal bar in the abscissa depicts different clusters of features according to their sensitivity, intensity selective features, contrast selective, intrinsic dimensionality features and symmetry sensitive features. (**B**) For each stimulus category (N, F, U and P), highest D_KL_ values for one-dimensional (leftmost bar) and two-dimensional (second bar from left) saliency functions are shown. The D_KL_ values obtained from the saliency functions of the interestingness maps are shown in the third place. Last bars represent highest possible D_KL_ value; these are obtained treating actual fixation maps as feature maps.

We found a main effect of low-level feature (F(64,14) = 283.25) and image category (F(64, 3) = 936.65) as well as a significant interaction between these two (F(64,42) = 65.3). Analyzing the data irrespective of image category (Fig. 4A, *gray* bars), correlations between fixation points and feature values were strongest with the feature set of Intrinsic Dimensionality (labels S, C and E). Among the three Intrinsic Dimensionality values, Cornerness had the highest D_KL_ values (D_KL_ = [.074 .105], square brackets denote 99.9% bootstrap derived confidence intervals) and Edgeness the lowest (D_KL_ = [.021 .031]) correlations. Furthermore, comparing Surfaceness and Edgeness, we found that in order to discriminate fixated vs. non-fixated points Surfaceness feature performed better than Edgeness feature (D_KL_ = .026 vs. .058). The second strongest feature was Red-Green Contrast (D_KL_ = [.06 .08]), which was not significantly different from Cornerness features (p>0.01) in this overall quantification. Within other features sensitive to contrast (**LC, YBC, TC, SATC**) we did not observe a significant difference (p<0.01) with the exception of Texture Contrast that had a relatively smaller D_KL_ value. Across the four features sensitive to first order statistics, **RGM** (D_KL_ = [.029 .041]) deviated significantly from the other features (**LM**, **YBM, SATM**). Among the phase symmetry features highest D_KL_ values of the radial symmetry feature (D_KL_ = [.021 .029]) were only marginally smaller than RGM. The lowest D_KL_ value was observed for the bilateral symmetry feature (D_KL_ = [.003 .005]).

The analysis of the main effect of image category revealed that D_KL_ values across different categories of images differed significantly. The strength of the correlation between feature and fixation points was strongest in the case of Urban (D_KL_ = [.059 .069]) and Fractal (D_KL_ = [.055 .066]) category. These two values were not significantly different from each other (p>0.01). This is reflected in the average saliency function computed across all tested feature channels (Fig. 3E). To compute these, we discarded the average Red-Green and Yellow-Blue hue channels, as the zero point within these feature channels is located at the middle of the scale. Additionally among the features of intrinsic dimensionality, we discarded Edgeness and Surfaceness as the Cornerness features makes the largest contribution to the description of the fixation locations. High D_KL_ values in the case of Fractal and Urban categories were caused by the steep increase of saliency as a function of feature percentile, due to the large deviations between image statistics at fixated and non-fixated locations. Surprisingly very small D_KL_ values characterized the overt behavior under Natural (D_KL_ = [.017 .021]) and Pink Noise (D_KL_ = [.017 .022]) categories (Fig. 3B). This shows that the correlation between low-level features and eye movements is subject to drastic modifications under normal viewing conditions.

Interestingly we observed a difference in the saliency curves between the Pink Noise and other category of images. The differences in the saliency functions of Fractal, Urban and Natural categories were mainly characterized by a modulation of the slope, but not by the sign of the saliency function. The saliency of a feature presented in these categories increased always monotonously with the feature percentile. This was however not true in the case of Pink Noise, where a monotonously decreasing relationship leading the less contrasted regions to be more salient. We therefore conclude that the saliency function associated to Pink Noise category is qualitatively different. Paradoxically, in the case of these images that are by definition described only by their second-order statistics, overt attention models based on the saliency of contrast features would not be expected to perform satisfactorily given that here low-contrasted regions are more likely to be fixated.

We observed a significant interaction between features and image categories (F(64, 42) = 65.3). In the case of Natural category, average Red-Green feature (D_KL_ = [.047 .074]) was the best predictor of fixation points and interestingly scored slightly higher D_KL_ values than Red-Green Contrast feature (D_KL_ = [.040 .054], Fig. 4A, *green* dots). The luminance contrast feature, considered typically to be a good predictor of fixation points, lead to surprisingly small D_KL_ values (D_KL_ = [.010 .014]) suggesting that color intensity is a much better predictor of fixation points. In the case of Pink Noise category, Red-Green Contrast (D_KL_ = [.010 .020]), Luminance Contrast (D_KL_ = [.021 .046]) and Surfaceness (D_KL_ = [.020 .040]) features were the best features in terms of their predictive strength (Fig. 4A, *magenta* triangles). With the only difference of Texture Contrast feature that scored low values in the case of Fractal, the rankings of features in the category of Urban and Fractal images were similar (*red* star and *black* squares). The best within these categories is the Cornerness feature (D_KL_ = [.140 .170] for Urban and D_KL_ = [.140 .180] for Fractal) that scored significantly better than all other features. These results suggest that the approach of finding a universal feature that has an overall validity is difficult. Indeed previous rapports have shown that the strength of correlations between fixation points and feature values changes with image category [49]–[51].

### Detection of Upper-limit

We reported D_KL_ values as a metric to quantify the correlation between features and fixation locations (c.f. [45]). In theory, this metric is bounded from both ends. On the one hand, the more similar the distribution of feature values at fixated and non-fixated locations are, the closer is the D_KL_ metric to zero. On the other hand, in the case of fixations concentrated in a very narrow range of feature values, the difference would be highest and the D_KL_ value would approach to the entropy of the distributions. However in practical terms this level of performance can never be reached because of continuous rather than binary nature of fixation probabilities. To evaluate observed D_KL_ values we therefore need to compute a practical upper bound. To this aim, we reasoned that a low-level image feature that would optimally detect fixated locations would need to be closely similar to the actual fixation maps. We therefore used actual fixation maps (averaged across all subjects, see Fig. 1 for an example of histogram equalized actual fixation map) as a substitute for the best hypothetical feature that would perfectly predict fixation behavior.

The D_KL_ values computed using fixation maps were very high and we obtained 0.69([.51 .91]), 1.06([.83 1.28]), 1.46([1.16 1.81]) and 0.39([.25 .59]) for the Natural, Fractal, Urban and Pink Noise categories, respectively (Fig. 4B, *fourth* bars in each category). Highest D_KL_ values obtained with low-level image based features (RGM for Natural and Pink Noise, Cornerness for Fractal and Urban) corresponded approximately to 10% of the highest D_KL_ values (Fig. 4B, first bars). The percentages were 12% for Natural, 7% for Fractal and 10% for Urban and Pink noise categories. This exemplifies the severe limitations of models that are purely driven by single low-level image-derived features. Still, we show here that within this small percentage there is a lawful relationship between feature values and their saliency, which exhibited (except in the case of Pink Noise) a monotonous relationship.

### Integration of Saliency

Models of overt attention working in parallel on multiple feature channels face ultimately the problem of integrating channel specific saliency information into a unique representation in form of a topographic map. This integration process is generally assumed to occur by a linear integration of individual saliency values. In order to elucidate how the saliency of different channels is integrated during free viewing of natural images, we computed two-dimensional saliency functions and modeled them by linearly integrating saliency functions of individual features.

**Figure 5:**
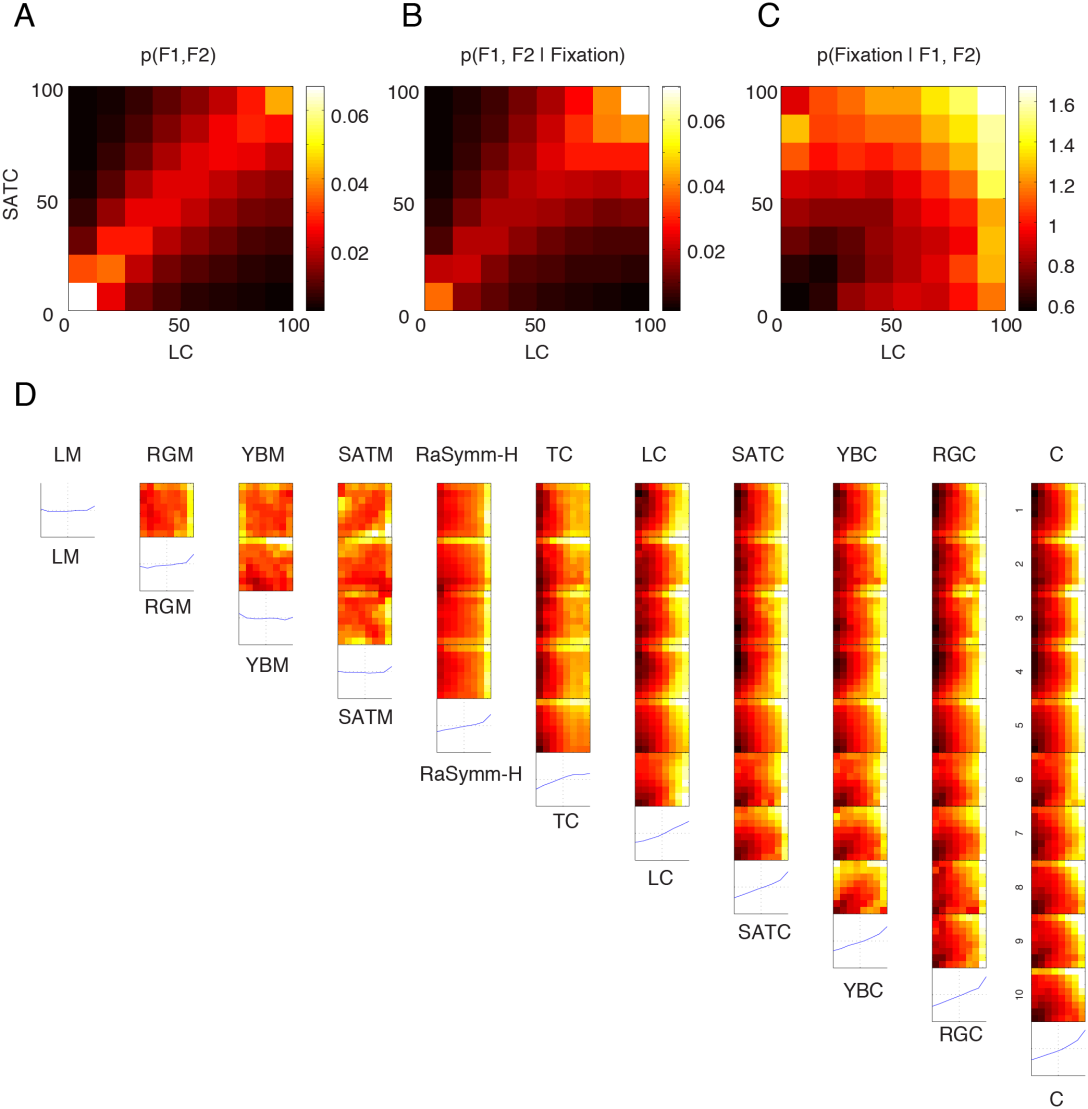
Computation of two-dimensional saliency maps. (**A**) Joint distribution of two example features F1 (luminance contrast, LC) and F2 (saturation contrast, SATC) is presented. This data doesn’t include Pink Noise category. Most of the points are located along the diagonal with a considerable accumulation of density at the lowest feature values. This joint distribution represents the co-occurrence of feature values at both actual and control fixation locations (including those that were not done on the shown image), consequently this joint distribution takes into account the central bias. (**B**) Distribution of the same feature pairs only at actually fixated locations. (**C**) The posterior probability distribution corresponding to the saliency function, p(fixation|F1, F2), is computed according to the Bayesian equality (see Materials and Methods). (**D**) Two-dimensional saliency functions are presented for a selected set of feature. The diagonal entries correspond to one-dimensional saliency functions presented in Fig. 3 but computed using only 8 bins. Features are ordered from left to right according to their D_KL_ values. To compute the saliency functions the data from Natural, Fractal and Urban categories were pooled. The data obtained during presentation of Pink Noise images was discarded. Abbreviations as presented in Fig. 1.

Intrinsic correlations between different feature channels within images make it difficult to test correlations between features at the fixated locations when more than one feature dimension is considered. Consider the situation depicted in Fig. 5A, which shows the joint distribution for two example features (Luminance Contrast and Saturation Contrast), p(Feature_1_, Feature_2_) at control and actual locations. Please note that although the marginal distributions are equalized as described above, the 2-D distribution is not homogeneous. Instead, due to the intrinsic correlation between these two features, pairs of feature values are mainly concentrated along the diagonal. In order to understand how these values are correlated with fixated locations we need to disentangle the intrinsic pair-wise correlations that exist in natural images from the distribution of feature values at fixated locations. As our Bayesian framework effectively takes intrinsic correlations into account, we can easily overcome this problem by computing the distribution of feature values at fixated locations, p(Feature_1_, Feature_2_|fixation) (Fig. 5B) and deriving from this the posterior distribution that represents the saliency function associated with these two features (Fig. 5C). The two-dimensional saliency function shows that the probability of fixation increases as a function of feature values along both feature dimensions and are highest at those image locations where both Luminance Contrast and Saturation Contrast are high simultaneously.

This calculation of a joint distribution was performed for each pair of features (11 different features, 99 different pairs) at control and actual fixations. We excluded the intrinsic dimensionality features except the Cornerness feature because the latter was the strongest feature in the single-feature analysis. Furthermore, in order to reduce the possibility of having bins without entries we reduced the number of bins to 8 per feature dimension keeping the total number of bins at 64 as in the one-dimensional case. Moreover, as the saliency functions of the Pink Noise conditions were qualitatively different than the ones taken from other conditions, we discarded them and performed the current analysis on the remaining 3 conditions by pooling all the data from remaining categories, i.e. we did not differentiate between different categories. The full set of two-dimensional saliency functions is shown in Fig. 5D. Here the one-dimensional saliency functions of each individual feature channel are computed at the same resolution and shown along the diagonal (Fig. 5D, diagonal panels). These functions are the low-resolution version of the saliency function that was studied in the previous section (Fig. 3).

**Figure 6:**
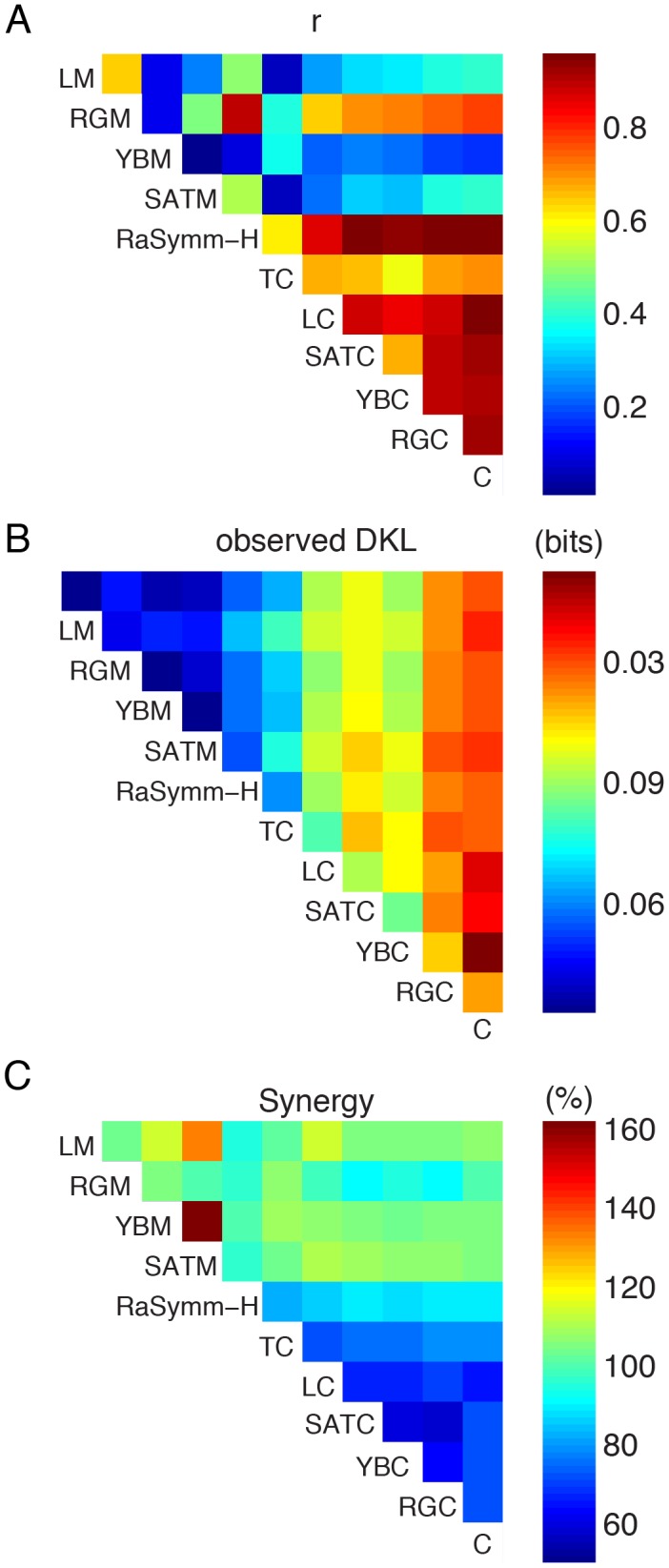
Integration of saliency functions. (**A**) The goodness-of-fit presented as a matrix for each pair of feature combination using a linear model for the data presented in Fig. 5D. Color codes for the strength of the correlation between empirical saliency maps (shown in Fig. 5D) and a model that linearly combines one dimensional saliency values. The ordering of the features follows D_KL_ values of one-dimensional saliency functions. Diagonal entries are omitted in this representation. (**B**) D_KL_ values extracted from two-dimensional saliency functions shown in Fig. 5D. Diagonal entries represents D_KL_ values of the one-dimensional saliency functions shown in Fig. 5D. (**C**) D_KL_ values of two-dimensional saliency functions shown in (B) are compared to the sum of corresponding uni-dimensional saliency functions (shown in the diagonal in (B)). 100% represents the case where the sum of the D_KL_ values of one-dimensional saliency functions equal to the D_KL_ value of the joint saliency function.

To test the hypothesis of independence, we modeled two-dimensional empirical saliency functions using the saliency of single channels, i.e. the marginals of the two-dimensional function, as independent variables. We found the best fitting regression coefficients and computed the variance explained using the squared correlation coefficients. The r^2^ values between the modeled and empirical saliency functions are shown as a matrix in Fig. 6A using the same configuration as in Fig. 5C. Mean r^2^ between predicted and empirical saliency functions was equal to 0.53([.44 .64], 99.9% CI). Adding an extra term that took into account multiplicative interactions between different feature channels improved the results only slightly (r^2^ = 0.54, [.45 .65], 99.9% CI). We therefore conclude that the bulk of the integration can be explained by independent contribution of different feature channels and therefore the assumption of different channels of features in models of overt attention is supported by our data.

Still, a careful inspection of the r^2^ matrix revealed the linear model of integration didn’t perform equally well for all feature pairs (Fig. 6A). The average r^2^ was 0.36[.28 .47] for all pairs that included only first-order features. This was much smaller than the r^2^ values of all other feature pairs, excluding the first-order features, where the average value of explained variance was equal to .82[.76 .90].

Next we elucidated whether the usage of more than one single feature would improve the discrimination of fixated locations from non-fixated ones. In order to quantify the incremental improvement of considering an additional feature we computed D_KL_ values between the p(Feature_1_, Feature_2_) and p(Feature_1_, Feature_2_|fixation) as analogous to the case of one-dimensional analysis. We computed the D_KL_ values for two-dimensional saliency functions shown in Fig. 5D in order to evaluate the strength of the correlation between pairs of features and fixated locations (Fig. 5B). Inspection of the D_KL_ matrix shows that D_KL_ values of 2D saliency functions were higher than the D_KL_ values of one-dimensional saliency functions (off-diagonal entries vs. diagonal entries in the Fig. 5B. Please note that the one-dimensional saliency functions are the low-resolution versions of Fig. 3, which obviously influences the computation of entropy and D_KL_ values.). In all cases D_KL_ values of two-dimensional saliency functions were higher than the highest D_KL_ value associated with one of the individual features.

In this image category blind quantification the combination of Cornerness and Red-Green Contrast features yielded the highest D_KL_ value. This demonstrates that considering more than one feature simultaneously has a positive effect in discriminating fixated locations from non-fixated ones. However, the increase of the D_KL_ value of 0.11 bits relative to the upper bound was only 18%. As the dominant feature was different for different categories, we next analyzed the two-dimensional saliency functions for each category separately and evaluated for each case the performance with respect to the upper bound. For the case of Natural stimuli the best combination of features that lead to highest D_KL_ values was Mean Red-Green and Red-Green Contrast features where a D_KL_ value of .061 bits was observed. For the category of Fractal and Urban, the same combinations gave the best results and D_KL_ values of 0.16 and 0.18 bits were obtained for the combination of Cornerness and Red-Green Contrast features. In the case of Pink Noise category, the strongest feature combination was Mean Red-Green and Luminance contrast features (D_KL_ = .034). These DKL values corresponded to 16%, 26%, 20% and 26% for categories of Natural, Fractal, Urban and Pink Noise, respectively (Fig. 3B). We therefore conclude that the incremental improvement by considering pairs of feature over single features on average only has a small effect.

In order to quantify this improvement, we compared D_KL_ values of the two-dimensional saliency functions to the sum of the corresponding D_KL_ values of one-dimensional saliency functions and expressed the difference as a percentage of predicted D_KL_ values (Fig. 5C). The observed D_KL_ values were most of the time smaller than the predicted D_KL_ values suggesting a sub-additive improvement in the discrimination of a fixated location from non-fixated ones. A synergistic supra-additive improvement occurred only at pairs of features where the mean luminance and mean saturation features were associated. These synergistic effects were mostly observed for feature pairs where each individual feature didn’t have high D_KL_ values. Furthermore these were also those pairs where the linear integration model didn’t give satisfactory results. This suggests that the stronger a feature pair was correlated with fixation points, the better a linear model explained this integration process. Therefore these results are compatible with a model of linear integration of low-level saliencies suggesting that no complex interaction schemes are needed in order to combine the saliency of different image feature channels.

### The Saliency of Behavioral Relevant Features

**Figure 7:**
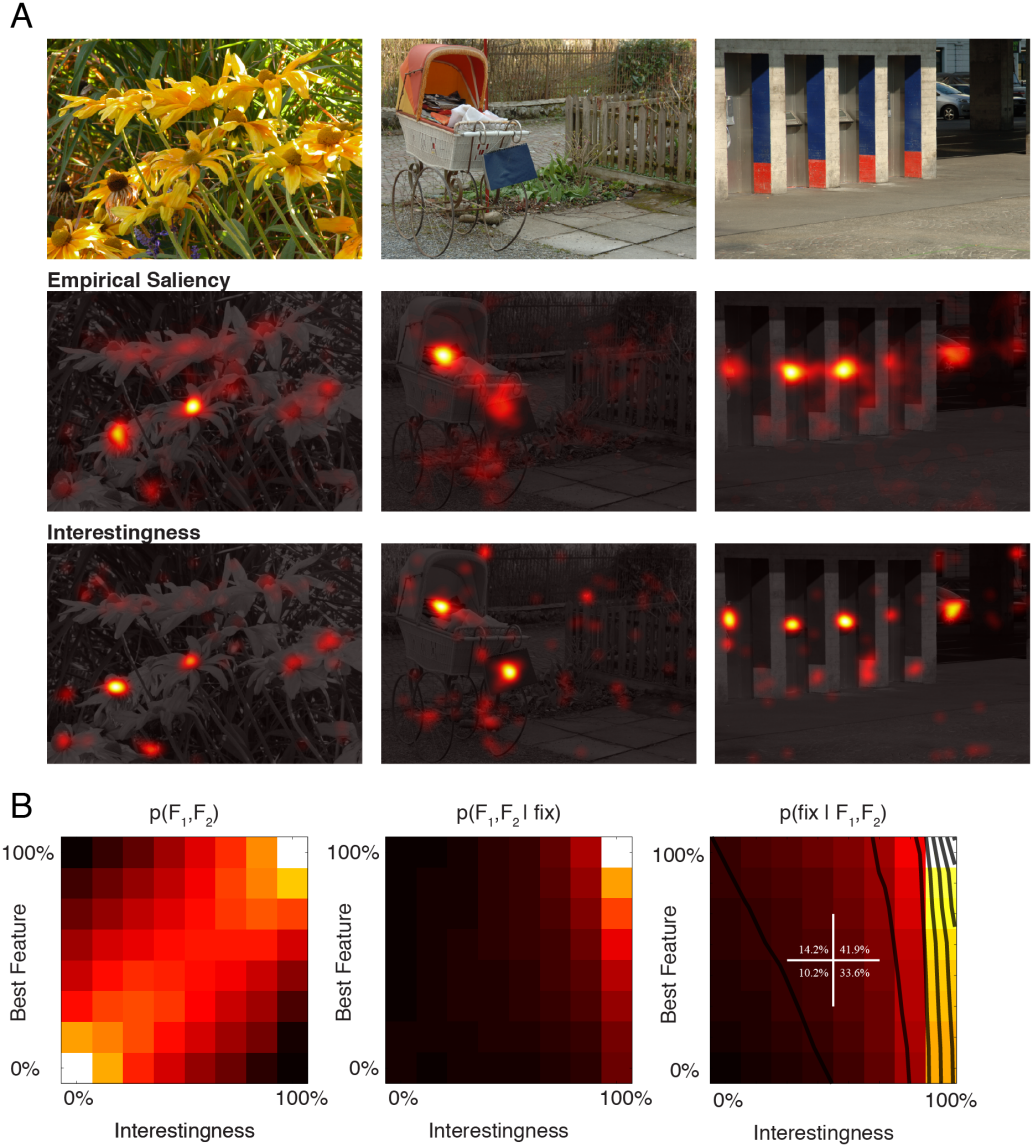
Computation of Interestingness maps. (**A**) 3 different stimuli are shown together with their actual fixation and interestingness maps (*second* and *third* rows). The first row depicts three stimuli belonging to Natural and Urban categories as they were shown during the experiment. *Second* and *third* rows depict the empirical saliency and interestingness maps overlaid on the gray scale version of the stimulus. Empirical saliency maps are probability maps that show the probability of a given location to be fixated. Similarly interestingness maps represent for a given location the probability of receiving an interestingness rating. These were obtained with the help of a pointer device by an independent set of human subjects (n = 35). These maps were treated the same way as low-level feature maps in order to compute D_KL_ values. (**B**) Joint distribution of interestingness values and a low-level feature (Cornerness) that was most strongly correlated with fixation locations (*Left* panel). This distribution shows the co-occurrence of a low-level image based feature with interestingness ratings of human subjects at all fixated and non-fixated locations. *Middle* panel represents the distribution of same variables at exclusively fixated locations. The posterior distribution represents the two-dimensional saliency function (*right* panel). Notice that the saliency increases nearly completely as a function of interestingness ratings (contour lines) and only marginally as a function of low-level feature values. The saliency is therefore mainly modulated by the interestingness value of a location.

Having quantified the correlation between attended image locations and their low-level attributes, we next focused on the question of to what extent a high-level feature would be correlated with selected fixation points. In order to quantify high-level content associated with a spatial location in an image we required an independent set of subjects (n = 35) to click on locations that they found interesting in an image with the help of a pointer device. We excluded the Pink Noise condition as these were devoid of any high-level information that needs to be processed. By accumulating all the click events for each single image (see Materials and Methods), we created maps that represented spatially the interestingness rates across subjects. Example stimuli (belonging to Natural and Urban) are depicted in Fig. 7A (*upper* row) together with the fixation and interestingness maps (*middle* and *lower* rows). Interestingness maps were very similar to fixation maps. However there were also notable differences between these two maps.

D_KL_ values derived from the saliency functions of interestingness feature were typically high and values of 0.28, 0.47 and 0.66 bits were obtained for the categories of Natural, Fractal and Urban. These D_KL_ values corresponded to 45, 48 and 41% of the estimated upper limit of D_KL_ values, respectively (Fig. 3B). Therefore independent of category of images, interestingness maps explained approximately a constant proportion of the fixation behavior. Most importantly, compared to the low-level features there was a drastic increase in the D_KL_ values, meaning that the interestingness ratings are a much better candidate for predicting locations of fixations than any other single or pair of low-level features.

Complex images such as those we used in this study are typically characterized by strong intrinsic correlations between different feature channels. For this reason, interestingness ratings that we here obtained might well be correlated with a hidden low-level image feature and therefore it is in principle possible that the subjective interestingness ratings include underlying low-level image features. – Such an argument is often put forward to question correlations of low-level features with high-level features that might truly determine selected fixation points. However, it works in both directions. – By constructing two-dimensional saliency functions of interestingness and another low-level feature we can dissect the contribution of these two and evaluate their relative contributions on the process of fixation point selection. We therefore computed two-dimensional saliency function of interestingness ratings and the best single low-level visual feature (Cornerness feature). The joint distribution of the best low-level visual feature and interestingness ratings was characterized by a strong correlation (Fig. 7B, left panel). This distribution shows how the feature values and interestingness ratings are distributed at control and actual locations. This correlation has been previously interpreted as the basis for bottom-up models to detect automatically interesting locations in an image [17]. When the joint distribution of interestingness and low-level features at fixated locations is considered, it is clearly visible that fixation points were preferentially directed at highly interesting locations (Fig. 7B, middle panel). Additionally, the probability density increased slightly with increasing low-level feature percentile. In order to compute the joint saliency function, p(fixation|Feature_1_, Feature_2_), we took the ratio of these two functions. The two-dimensional saliency function shows that the saliency was to a large extent modulated by the level of interestingness (see parallel contour lines) and saliency increased steeply with increasing interestingness. This indicates independent contributions of the individual low-level feature (here Cornerness) and high-level feature (interestingness).

These results show that the interestingness rating of that location largely determines whether a location is to be fixated or not, while the contribution of low-level image features to the process of selection is significant but smaller. In order to further quantify this, we divided the saliency function (Fig. 7B right panel) into 4 quarters representing all combinations of weak and strong high/low-level saliency, and computed the accumulative saliency within each quarter. The accumulative saliency of regions with weak feature intensity and interestingness rates was small (10.2%). Locations with weak interestingness ratings but which had nevertheless strong low-level feature values were only slightly more salient (14.2%). However, those locations with weakest low-level feature values, which were however judged to be very interesting, were approximately three times more salient (33.6%). The incremental effect of an increase in the low-level feature level for those locations that were rated interesting was 8.3%, leading to an accumulated saliency of 41.9% for those locations with strong low-level values and interestingness ratings. Therefore, the low-level image features had a significant effect, and this was strongest for those points where the interestingness ratings were already high.

We fitted the following function, α·Interestingness + β·Feature = Saliency, in order to quantify the contribution of different independent sources of information. A simple linear model without an interactive term accounted relatively well the observed integration (r^2^ = 0.63). Importantly, the relative contribution of Interestingness feature was 4.57 times stronger than the best low-level feature (α = 0.28; β = 0.06). Including an interaction term in the form of γ·Feature·Interestingness, didn’t have a major impact on the quality of the model (r^2^ = 0.64). Because of the steep increase in the saliency function, an expansive non-linearity, α·Interestingness^x^ + β·Feature^x^ improved considerably model prediction. For x = 2 and x = 4, we obtained r^2^ = 0.77 and 0.91; inclusion of a term that took into account interaction had again only minor effect (r^2^ = 0.79 and 0.93). Our results suggest that interestingness ratings do operates nearly independently of low-level image feature and have the best predictive power relative to single low-level features for the detection salient locations in an image.

### Time Course of Low-level and High-level Contributions

It is important to understand how fast these interesting locations are detected by human subjects under free viewing conditions and especially how this detection performance relates to the temporal course of low-level feature vs. fixation correlations. In one scenario detection of interesting points could require some time. According to this view subjects would need to scan the picture completely before detecting what is interesting in an image. This scheme matches the instruction of the second cohort of subjects, marking interesting locations in the images. The predictive power of interestingness, and therefore the D_KL_ values would then display an increasing trend along the stimulus presentation time. Alternatively, the information concerning the location of interesting points could be quickly available to human subjects based on the gist of the scene, and fixation points could initially be directed at those locations in order to extract the most relevant information. Subsequently, details and low-priority locations could be selected to complement the processing of the image. These two hypotheses predict very different dependencies of the D_KL_ values on the presentation duration.

**Figure 8:**
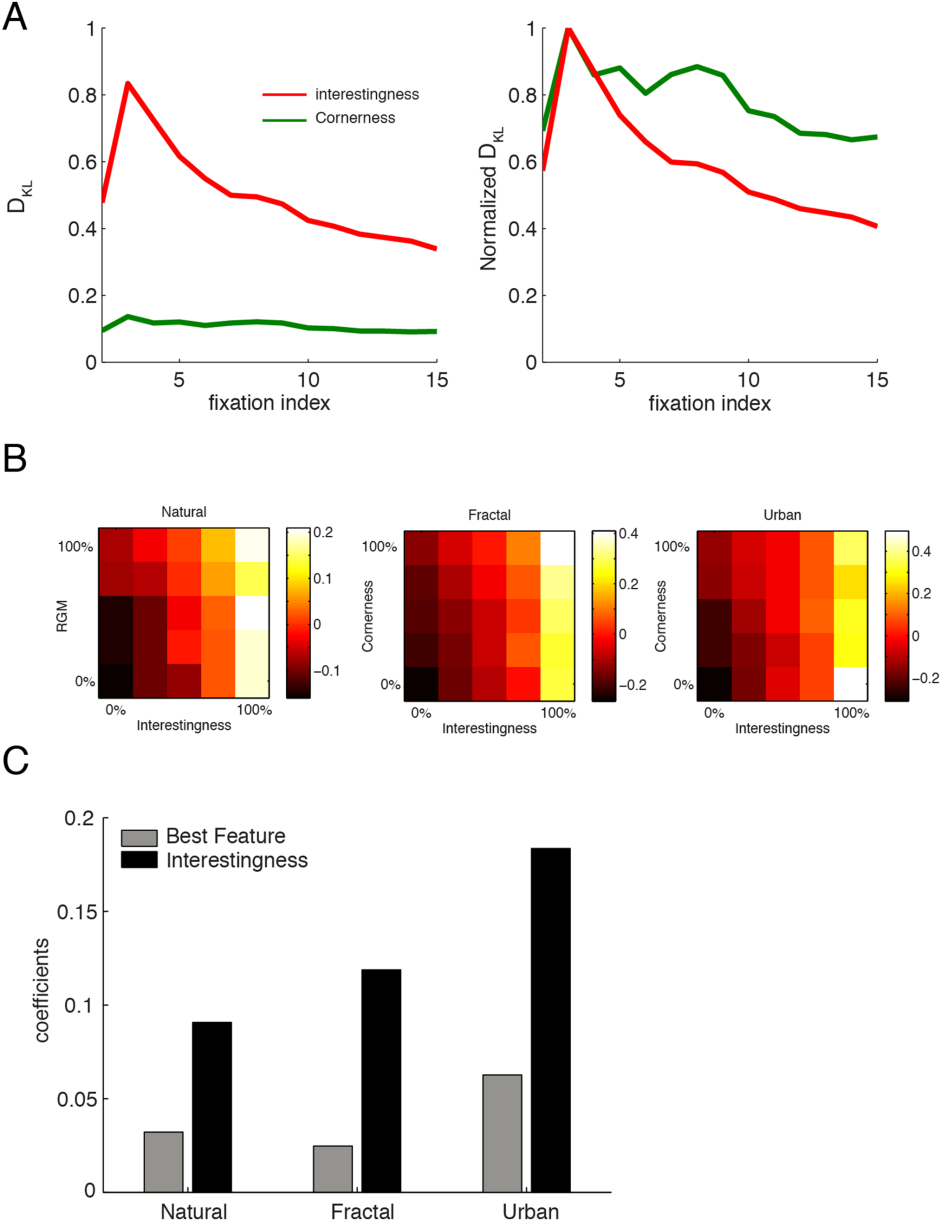
(A) Time course of D_KL_ values for high- and low-level features; these were computed for each fixations separately starting from the second fixation on. Cornerness feature was used to illustrate the time-course of a low-level feature. While, absolute D_KL_ values *are* shown in the *left* panel, the curves were normalized to their peak value (second fixation) in order to have a better comparison of the temporal evolution. (**B**) Joint distribution of fixation durations computed separately for Natural (*left* panel) and Urban (*right* panel) categories. (**C**) For different image categories, each bar represents the contribution of low- and high-level information on the variability of duration of fixations.

To investigate these hypotheses, we computed D_KL_ values for each fixation separately (Fig. 8A, *red* lines). During these calculations, the control and actual fixation maps were accordingly modified. We observed that the D_KL_ values peaked as early as 3^rd^ fixation and subsequently decreased monotonically and reached values approximately 1/3 of peak values. We conclude that locations, which were rated interesting, are as early as within the first second of stimulus presentation detected and the scanning behavior following this initial scanning shifts towards less interesting regions. Therefore the detection of interesting locations do not occur at a glance, i.e. with the very first fixation, but still surprisingly early after stimulus onset.

The D_KL_ time-course in the case of low-level image characteristics could in principle be independent of the D_KL_ value of interestingness and may follow increasing, decreasing or a constant trend. A decreasing trend would be an evidence for a fixation behavior that initially selects locations with strong low-level feature values, and later, locations with weak low-level saliency. We computed the time-course of D_KL_ values for Cornerness feature which had the highest D_KL_ values (Fig. 8A, *green* lines). In the left panel, the time-course of absolute D_KL_ values are shown, because of the large difference of D_KL_ values the precise structure of D_KL_ values of low-level features are not well resolved. In the right panel these data are separately normalized to the peak value of each curve in order to allow a better comparison. Time-courses were alike and all characterized with a decay starting from the third fixation. However, the initial rise and later decay is smaller in the case of Cornerness feature as compared to the Interestingness.

### Analysis of Fixation Duration

It is a well-established fact that not all fixations last equally long. Yet this fundamental property of eye movements is often neglected in studies of overt attention and no model of overt attention so far does account for this variability [4]. Average fixation durations varied considerably across both subject (M = 302 ms, S = 55 ms, Kruskall-Wallis Test, Chi-2_47_ = 9372, p<10^−5^) and categories (Kruskall-Wallis Test, Chi-2_2_ = 544.1, p<10^−5^). Fixation durations for the Urban category was approximately 9% significantly shorter than the fractals and naturals (312 and 310 ms, respectively), corresponding to 26 ms +/−17 ms (t-test, p-value = 0.0024), there were no other differences between different categories.

It is also important to understand the variability at the single subject level. This variability originates in a number of sources, e.g. the saccadic momentum [52]. We next assessed how fixation durations correlate with low- and high-level image characteristics respectively. And we computed the fixation durations jointly as a function of different percentile of interestingness and best low-level feature (Mean Red-Green for Natural and Cornerness for Fractals and Urban). This was done for all three categories where we recorded interestingness ratings (Fig. 8B; *left* Natural, *middle* Fractal, *right* Urban). However, in order to discount the large variability of fixation durations between subjects, fixation durations were z-score transformed beforehand for each subject, this was done for each category separately. This allowed us to disregard inter-individual differences, and focus on how fixation durations changed as a function of low- and high-level image values.

These figures show a clear effect of interestingness on fixation durations and a less pronounced impact of low-level feature values. As in the previous case, we computed the regression coefficients in order to compute the contribution of different factors (Fig. 8C). The quality of the fits was higher than 0.9 in all cases. Whereas the contribution of interestingness was, as detected by the regression analysis, 0.09, 0.11, 0.18 for Natural, Fractal and Urban categories, these values were equal to 0.032, 0.02, 0.06 for the best low-level feature. Therefore the impact of interestingness was about 2.8, 4.8 and 2.9 times stronger than best single low-level feature. These results show that the variability in the fixation durations can be accounted by the interestingness ratings of different image locations and to a lesser extent by the low-level structure of the images.

## Discussion

Eye-movements, the reliable mirror of attentional allocation, are second nature to humans and many other animals. Undoubtedly many different sources of information contribute during this process [3]. Bottom-up attentional allocation results from the elements of a scene directly attracting the spotlight of attention [53]. This relates to a whole hierarchy of visual features that may be combined linearly or non-linearly [34], [54]–[56]. Additionally, attentional resources can also be directed in a top-down manner by the task and meaning of different image components [2]. This relates to people, objects, their configurations and relationships etc. Whether these two processes draw on joined or separate mechanisms is an issue of current debate [32]. Indeed, recent evidence from lesion and TMS studies indicate the parallel operation of distinct attentional systems, one of these being well described by a saliency map [57], [58]. Furthermore, the relative contribution varies with age in the form of a high contribution of stimulus-dependent information at young age and a reduced contribution in elderly subjects [59], [60]. It is clear that everything that adds a value to the visual scene on the eye of the active observer attracts fixations, as it is the case in the presence of an explicit task. Current models of overt-attention founded on the concept of saliency are either limited to the former contribution or are based upon the assumption that these two information sources co-occur in a visual scene, that is salient low-level aspects of a scene are de facto those that are also significant and meaningful to an organism.

The present study starts with a decision which visual features to include and which not to include [61]. In previous studies a large number of features has been shown to correlate with visual saliency to varying degrees: luminance contrast [11], texture contrast [48], [62], edges [63], color contrast [50], [64], intrinsic dimensionality [40], symmetry [65], disparity and disparity contrast [15], [66], motion [67]–[69], faces and text elements [70], [71]. Of these, the latter three were not compatible with the design of the study. Disparity had to be excluded, as the images were not available in stereoscopic pairs. Presenting videos would lead to one fixation per subject on each frame and hence to highly sparse data. As a consequence, the analysis as performed here would not have been possible. Furthermore, although motion is a salient cue [67], recent studies compared eye movements of actively moving observes to eye movements under lab conditions with videos or still images presented [68], [72]–[74]. Although motion emerges as an important cue it is not a critical factor in models of visual saliency. Another study reports the high saliency of faces and text elements [70]. However, in the present study neither individual faces nor text elements were numerous in the stimulus set. Considering these boundary constraints we tried to compile an as inclusive list as possible. Furthermore, an earlier study Kienzle et al. [75] reports optimal features being characterized by a center surround organization. This is reminiscent of receptive fields observed in the early visual system and can be conceptualized as luminance contrast detectors at specific spatial frequencies. This argues that the selected set of image features captures the majority of relevant low-level cues and that there is no yet unknown feature to be discovered that will dramatically tilt the balance in favor of the low-level features.

A considerable amount of clicks were directed at the middle of the tunneling patterns of fractal images. Somewhat similar to this observation, during viewing of natural scenes many fixations were accumulated at the center of the circular patterns such as for example flowers, which were also rated interesting. This suggests that the symmetry features play an important role. We tested 2 different symmetry features, even though Radial symmetry feature lead to relatively high D_KL_ values, overall their contribution was limited. Recently in a report dedicated to the saliency of symmetrical configurations, Koostra et al. [65] proposed that symmetry features are as important as luminance contrast derived features and should also be included in the implementation of saliency models.

In order to control for differences in units and properties of image features we use a histogram equalization scheme. Different features come in varying units, posing the question of a natural scale for comparison. Worse, non-linear scaling of a feature has a large effect qualitatively changing its contribution in a linear combination of all features. As a consequence, investigations of properties of integration of different features [34], [55], [56] depend on the chosen scale. For example, is the standard deviation or the variance (square of standard deviation) of luminance in a local region the better measure for luminance contrast? In case the linear combination of the variance of luminance and red/green axis is a good model of saliency, then the linear combination of the respective standard deviations is not necessarily a good model. This problem may be addressed by machine learning techniques [76]. The histogram equalization scheme proposed here is invariant under monotonous warping of a feature. This takes into account all the peculiarities of different feature maps and equalizes the distribution of all values to a uniform distribution. Hence it is a natural choice for the scale and unit of features contributing to a model of saliency.

Previous studies report a qualitative influence of context and image class on the allocation of overt attention [7], [22], [50]. We used 4 different categories (Natural, Urban, Fractal and Pink Noise) of images and quantified eye-movement behavior in the absence of a specific task. Using a Bayesian approach we characterized the relation of low-level and high-level stimulus based information sources, their saliency and integration. Out of the 4 stimulus categories used exploratory eye-movements on Natural Images and Pink Noise images yielded comparable results. Total coverage of individual images, intersubject similarity, the category specific fixation patterns were alike within these two sets of categories. Here, the total coverage was small and the central bias had a large impact, reaching high levels during viewing of pink noise images. Exploration characteristics of Urban and Fractal images were similar to each other. Steeper saliency functions hence a stronger correlation between fixations and low-level features were observed in the case of fractal and urban images yielding a moderate total predictive power. This held even when multiple features were taken into account. These results demonstrate and extend previous reports of largely varying properties of the spatial bias and predictive power of low-level stimulus features as a function of image category [50].

The grouping of Fractal and Urban on the one hand and Natural and Pink Noise images is an interesting one and similar grouping was also observed for the analysis of exploration strategies. Fractal images are rich in low and mid-level information, however in contrast to urban sceneries, they are completely devoid of any meaning for that common objects and people characteristic of natural and urban scenes do not exist in fractal images. On the other extreme, pink noise images are perfectly defined by second-order statistics and neither semantic nor high-level image features are present. Human subjects were consistent in their interestingness judgments for fractal images; suggesting that even at the absence of objects as encountered in an urban or natural sceneries humans can subjectively find interesting locations consistently. Furthermore, in those categories of images where the inter-subject correlation was high (such as fractals and urban) the correlation between fixations and feature values were found to be high. In contrast, for those images where the fixation patterns were dissimilar between subjects (natural and pink noise), the dependence on features was also weaker. We conclude that different category of images, not only induce different spatial fixations patterns resulting in different exploration strategies, but also simultaneously in different feature-fixations correlations, suggesting that these two are tied together.

We find that the strength of different features is noticeably different for different image categories. This emphasizes the impact of context or image gist in the allocation of attention. Furthermore, in the case of Fractal and Urban category, Cornerness feature was the most predictive feature, and Red-Green Luminance intensity was best for the Natural and Pink Noise. It is notable that within these two categories the raw intensity, rather than the contrast was more strongly correlated with fixations. We also note that saliency functions in the case of pink noise category of images were qualitatively different, in the sense that they were not monotonously increasing functions of feature intensity. This demonstrates the problems to find a feature set that has an overall validity is difficult. Interestingly, recent studies demonstrate the impact of contextual information on object recognition [77], a process thought to follow the allocation of attention. This suggests that the predictive power of a general stimulus driven attentional mechanism should integrate contextual information in order to achieve satisfactory performance.

The analysis of fixation durations uncovered significant differences between image categories. Fixations on urban images, where the predictive value of features was high, fixation durations were shorter than in the other image categories. This demonstrates that in the case of a rich image structure exploration is not necessarily slowed down to allow in depth processing. Instead, the competition to scan other image regions might more than compensate such an effect and lead to reduced fixation durations. We have to note however, that the predictive power of stimulus features is high in the case of fractals as well, yet the fixation durations are noticeably longer. Hence, the current data do not allow the formulation of a mechanistic model of fixation duration and this must be left for future work.

We resorted to interestingness ratings of an independent set of human subjects collected with the help of a pointer device and derived topographical maps of interestingness. Here, the correlation levels as measured with D_KL_ metric were very high. This suggests a strong contribution of semantic processing to guidance of eye-movements and in turn limiting the contribution of low-level characteristics of the image. These two sources of information, namely low-level and high-level features were estimated to have independent impacts on the eye-movements with a low-level of integration.

The interest in the role of high-level aspects of a scene in guiding eye-movements is not recent and many reports aimed to characterize the influence of high-level scene content on overt behavior under free viewing or different task conditions [26], [78]. One of the most used high-level metric is the semantic congruency of objects located in a visual scene [27], [28], [30]. For example by including an object, which would normally not be located at a given context (e.g. a car on a sofa), the effect of high-level factors on allocation of attention can be characterized. The detection performance of such incongruent targets in a change blindness paradigm, duration of fixations as well as the position of fixation points seems to be modulated by these high-level factors [29], which is in accord with the results presented here. However during such experimental paradigms, the precise evaluation of semantic incongruency is not parametric, difficult to quantify and depends on the experimenter’s judgment.

Interestingly the upper-limits for D_KL_ values were different for each category of images and the way in which the upper bound depended on the category was very similar to the inter-image agreement as quantified by the entropy of the category specific feature maps (compare fourth bar in Fig. 4B and dashed line in Fig. 2A, Please note that low entropy implies high inter-image similarity here.). This similarity can be explained by the fact that when inter-image agreement is high (as in the case of pink noise images), the similarity between the category specific and image specific maps should necessarily be high also. This in turn influence the maximum D_KL_ values that can be obtained given that feature distributions at fixated points are very similar to control points. In fact, the upper maximum D_KL_ value is a good measure of how much image-specific behavior there exist to be explained. As expected, the behavior during Pink noise images being rather stereotypical, there is not much image-specific information that is to be explained. Furthermore this also justifies the use of a practical rather than a theoretical upper bound.

In our report, we used a direct evaluation method by collecting interestingness ratings of human subjects with the help of a pointer device. Interestingness is a handy concept that summarizes well high-level aspects associated to different portions in an image. This technique, similar to Antes et al. [30] and more recently to Masciocchi et al. [36], results in two-dimensional maps of interestingness. Similar to low-level image features, these maps provide topographic distribution of image locations with high-level significance. We showed that interestingness ratings of human subjects correlated well with fixation locations. Locations with high interestingness ratings are found to be highly salient and efficiently attract eye-movements. Specifically, although subjects had unlimited time to explore the visual stimuli, the geometrical properties of saccades to salient points and the distribution of interesting points are very similar. Furthermore, we found that humans are able to detect these interesting spots in a scene relatively quickly after the stimulus onset. Overall these results show that high-level aspects of a scene as capture by the interestingness metric are highly efficient in guiding eye movements.

Recently, the position of objects in a visual scene [26], the informativeness [5], and the interestingness ratings [36] have been used to empirically derive topographic maps of high-level information directly from a scene. Einhäuser et al. [26] used a model taking into account exclusively the position of objects to predict fixation positions. This model performed much higher hit rates than a model considering only low-level attributes [26]. Similarly, Kollmorgen et al. [5] measured the contribution of low-level features (stimulus-dependent information), informativeness (task dependent information) and localization (spatial constraints) to the guidance of eye movements in four different tasks. They report a consistent and high contribution of the spatial properties. The influence of task dependent information was more variable and slightly lower. Stimulus-dependent properties had a still smaller and variable, but nevertheless significant contribution. Importantly, the contribution of the three different types was largely independent. Furthermore, Einhäuser et al. [78] report that in complex visual stimuli task demands may override stimulus driven saliency almost immediately. These results indicate that the question “Are low-level stimulus-dependent features or high-level task dependent information more important in guidance of eye movement?” might be ill posed and be denied of a simple one-dimensional answer [79].

It is certain that the saliency based approach to overt-attention and its elaborated versions provide a clearly defined formal scheme for the investigation of eye movements, and still today provides a major impetus to the field of overt attention. Furthermore, the saliency model and its derivatives are currently used as the benchmark model upon which different hypotheses can be tested. Already early on it has been argued in favor of complementary systems on one hand supporting focus on the task at hand and on the other hand switching attention to unexpected potentially relevant events. Further exploration of this relation will deepen our understanding of this central mental ability as well as contribute to the development of helpful technical artifacts [80]. The concept of a saliency map thrives on stimulus-dependent information and in its most common form is ignorant on the task context. Hence, it is best suited to model the latter, purely stimulus-dependent component. This, however, does not imply that it needs to incorporate only low-level image properties. Instead, including progress of our understanding of high-level image features (objects) and modulation of contextual information (image categories) are expected to further improve models of the stimulus-dependent component of overt attention.
